# Longitudinal Associations of Multimodal Core 1 Alzheimer Disease Biomarkers With Cognition in Aging and Preclinical Alzheimer Disease

**DOI:** 10.1212/WNL.0000000000214308

**Published:** 2025-10-17

**Authors:** David López-Martos, Marc Suárez-Calvet, Marta Milà-Alomà, Juan Domingo Gispert, Gemma Salvadó, Anna Brugulat-Serrat, Mahnaz Shekari, Carolina Minguillon, Henrik Zetterberg, Kaj Blennow, Oriol Grau-Rivera, Gonzalo Sánchez-Benavides, for the ALFA study, Federica Anastasi

**Affiliations:** 1 Barcelonaβeta Brain Research Center (BBRC), Pasqual Maragall Foundation, Spain;; 2 Hospital del Mar Research Institute, Barcelona, Spain;; 3 Centro de Investigación Biomédica en Red de Fragilidad y Envejecimiento Saludable, Instituto de Salud Carlos III, Madrid, Spain;; 4 Servei de Neurologia, Hospital del Mar, Barcelona, Spain;; 5 Department of Veterans Affairs Medical Center, Northern California Institute for Research and Education (NCIRE), San Francisco;; 6 Department of Radiology, University of California San Francisco;; 7 Clinical Memory Research Unit, Department of Clinical Sciences Malmö, Lund University, Sweden;; 8 Global Brain Health Institute, San Francisco, CA;; 9 Department of Psychiatry and Neurochemistry, Institute of Neuroscience and Physiology, University of Gothenburg, Mölndal, Sweden;; 10 Clinical Neurochemistry Laboratory, Sahlgrenska University Hospital, Mölndal, Sweden;; 11 Department of Neurodegenerative Disease, UCL Institute of Neurology, London, United Kingdom;; 12 UK Dementia Research Institute at UCL, London, United Kingdom;; 13 Hong Kong Center for Neurodegenerative Diseases, Clear Water Bay, China;; 14 Wisconsin Alzheimer's Disease Research Center, University of Wisconsin School of Medicine and Public Health, University of Wisconsin-Madison;; 15 Paris Brain Institute, ICM, Pitié-Salpêtrière Hospital, Sorbonne University, Paris, France; and; 16 Neurodegenerative Disorder Research Center, Division of Life Sciences and Medicine, and Department of Neurology, Institute on Aging and Brain Disorders, University of Science and Technology of China and First Affiliated Hospital of USTC, Hefei.

## Abstract

**Background and Objectives:**

Addressing the association between early-changing Alzheimer disease (AD) biomarkers and cognition is essential for characterizing preclinical AD. However, few studies have explored this relationship longitudinally, especially across multiple biomarker modalities. The aim of this study was to evaluate longitudinal associations of multimodal core 1 AD biomarkers with cognition in a cognitively unimpaired (CU) population at increased risk of AD dementia.

**Methods:**

This prospective observational study included CU individuals from the Alzheimer's and Families+ cohort, based in Barcelona, Spain. Individuals had available baseline CSF biomarker measurements (normal or AD *continuum* profiles) and longitudinal neuropsychological assessments (2 time points, 3-year follow-up). The primary study outcome was the modified Preclinical Alzheimer's Cognitive Composite (mPACC) score. Study measurements included plasma phosphorylated tau (p-tau) 217, CSF p-tau181/β-amyloid (Aβ) 42, and Aβ PET ([¹⁸F]flutemetamol). Mixed-effects models were used to evaluate the longitudinal associations between fluid biomarkers and mPACC score; voxel-wise models were used to evaluate the longitudinal association between neuroimaging and mPACC score. Sensitivity analyses evaluated these associations stratifying by Aβ status.

**Results:**

In total, 350 individuals were included (mean age: 61 years; 60% female; mean education: 14 years). In the full sample, increases in plasma p-tau217 concentration (β_STD_ = −0.124, 95% CI −0.172 to −0.076; *p* < 0.001), CSF p-tau181/Aβ42 ratio (β_STD_ = −0.059, 95% CI −0.100 to −0.019; *p* = 0.005), and Aβ PET load in the prefrontal cortex, cingulate cortex, precuneus, and anterior temporal regions were associated with mPACC decline. In the Aβ-positive group (35%), increases in plasma p-tau217 concentration (β_STD_ = −0.121, 95% CI −0.194 to −0.049; *p* < 0.001) and Aβ PET load in anterior temporal regions were associated with mPACC decline. In the Aβ-negative group (65%), increases in plasma p-tau217 concentration (β_STD_ = −0.084, 95% CI −0.157 to −0.011; *p* = 0.024) and Aβ PET load in frontoparietal regions were associated with mPACC decline.

**Discussion:**

This study demonstrated significant longitudinal associations between multimodal core 1 AD biomarkers and cognitive function over 3 years in CU individuals, including those at the Aβ-negative stage. Distinct biomarker modalities provided complementary insights, emphasizing the potential of blood-based biomarkers for tracking subtle cognitive decline and neuroimaging for delineating vulnerable brain regions in aging and preclinical AD. These findings are crucial for identifying and monitoring high-risk CU individuals, further informing preventive intervention strategies for AD.

## Introduction

Alzheimer disease (AD) is a progressive neurodegenerative disorder that advances along a *continuum*, beginning with an extended preclinical stage, progressing to mild cognitive impairment (MCI), and ultimately leading to dementia. The accumulation of β-amyloid (Aβ) plaques and tau tangles in the brain defines the core biology of AD. In 2018, the National Institute on Aging (NIA) and the Alzheimer's Association introduced a research framework based on the status of Aβ (A), tau (T), and nonspecific neurodegeneration ([N]) biomarkers, establishing a classification system to characterize the AD *continuum* through AT(N) biomarker profiles.^1^ This framework was revised in 2024 by the Alzheimer's Association, distinguishing 3 major biomarker groups: (1) core AD biomarkers (i.e., Aβ and tau), (2) biomarkers of nonspecific processes involved in AD pathophysiology (i.e., neurodegeneration and inflammation), and (3) biomarkers of non-AD copathology (i.e., vascular brain injury and alpha-synucleinopathy).^2^ Within core AD biomarkers, a further distinction was made between diagnostic early-changing core 1 AD biomarkers—including Aβ PET, Aβ and tau CSF hybrid ratios, and plasma tau biomarkers—and prognostic late-changing core 2 AD biomarkers—particularly, tau PET. Early in the AD *continuum*, pathologic levels of these biomarkers can be detected before clinical onset, demonstrating consistent associations between AD pathology and cognitive function in cognitively unimpaired (CU) population.^3-5^ However, the conceptualization of AD at this early stage remains a topic of ongoing debate, particularly concerning the appropriate classification of CU individuals with positive AD biomarkers but no clinical impairment. The framework proposed by the Alzheimer's Association suggested to classify these individuals as being in the “preclinical stage of AD”^1,2^ while the framework proposed by the International Working Group suggested instead referring to them as being “at risk of AD,” because it is uncertain whether, or when, clinical impairment will ever manifest.^6^ Regardless of these differing perspectives, current research remains focused on uncovering the clinical-biological trajectory of AD, with the final goal of advancing early detection and prevention strategies.^7^

The Alzheimer's Association introduced the concept of transitional decline in relation to the preclinical stage of AD, delineating the shift from normal cognition to the subclinical manifestations that precede the onset of clinical impairment in the AD *continuum*.^1,2^ The guidelines for diagnostic recommendations consider that transitional decline can be identified by longitudinal testing, demonstrating subtle cognitive and/or behavioral decline, whether objective (documented by the clinician) or subjective (reported by the individual or an informant). Specifically, subtle cognitive decline can be objectively identified using standardized neuropsychological assessments, underscoring the relevance of the preclinical stage for identifying those individuals at higher risk of clinical progression.^8,9^ Early in the AD *continuum*, subtle cognitive decline has been primarily associated with lower performance in episodic memory and executive function, among other cognitive domains.^10,11^ The Preclinical Alzheimer's Cognitive Composite (PACC) and its modified version for optimization (mPACC) are particularly sensitive to Aβ pathology because they incorporate key neuropsychological measures from these distinct cognitive domains.^12,13^ Both PACC and mPACC have been established as primary end points in AD clinical trials,^14,15^ underscoring the importance of using sensitive neuropsychological methods for accurately tracking subtle cognitive decline in relation to AD progression. With emerging disease-modifying treatments for AD and further advancements anticipated, the potential benefits of early intervention strategies, such as those targeting Aβ reduction in asymptomatic individuals, are increasingly recognized.^7^ To optimize treatment efficacy, it is essential to identify high-risk individuals at the optimal disease stage for intervention. Recent advances in plasma biomarkers provide relevant opportunities in the field, allowing early detection and monitoring of AD pathology through minimally invasive blood sampling.^16,17^ Despite these advancements, most research on subtle cognitive decline has relied on cross-sectional biomarker data and only a few studies have addressed the longitudinal association between changes in biomarkers and the corresponding neuropsychological changes in the CU population.^18^ This gap limits the understanding of clinical-biological dynamics in AD. Beyond cross-sectional associations, the longitudinal evaluation of AD biomarkers in relation to cognition offers a clearer view of disease progression by tracking early changes in the disease trajectory over time, a crucial approach for improving characterization of preclinical AD.^19,20^ A review and meta-analysis found that, among the few available longitudinal studies, findings have been inconsistent, with only a small subset reporting significant effects. Remarkably, substantial heterogeneity was observed in both the biomarker modalities used to assess early AD pathologic changes and the neuropsychological tests used to measure cognitive function.^21^

To address this gap, this study examined the longitudinal association of multimodal core 1 AD biomarkers—including plasma phosphorylated tau (p-tau) 217, CSF p-tau181/Aβ42, and Aβ PET ([¹⁸F]flutemetamol)—with cognition, assessed using the mPACC, over a 3-year follow-up period in CU individuals at increased risk of AD dementia.

## Methods

### Study Participants

This study was conducted within the Alzheimer's and Families+ (ALFA+) cohort, a longitudinal study nested in the Alzheimer's and Families (ALFA) parent cohort at the Barcelonaβeta Brain Research Center, Barcelona, Spain.^22^ The ALFA cohort enrolled CU individuals aged 45–74 years with no significant medical, psychiatric, or neurologic conditions. The ALFA+ cohort included a subset of participants from ALFA, selected based on AD risk profile considering parental AD history and APOE-ε4. Inclusion in ALFA+ required previous ALFA participation, age 45–65 years, and willingness to complete all procedures (clinical and neuropsychological assessment, blood and CSF sample collection, and MRI and PET acquisitions). Individuals with cognitive impairment, systemic illness, or a family history of monogenic AD were excluded. Full details are available in eMethods.

In this study, we included participants from the ALFA+ study. Individuals were CU with available baseline (BL) CSF biomarker measurements and longitudinal neuropsychological assessments (2 visits over a 3-year follow-up), representing the subset with complete data across the minimum required dimensions for these analyses. Individuals with normal CSF biomarker profiles or falling within the AD *continuum* were retained for analysis and those classified as having suspected non-AD pathology were excluded, as defined by the AT(N) framework. A flowchart illustrating the participant selection process is presented in eFigure 1.

### Standard Protocol Approvals, Registrations, and Patient Consents

The ALFA+ study (ALFA-FPM-0311) was approved by the Independent Ethics Committee of Parc de Salut Mar, Barcelona, and is registered on ClinicalTrials.gov (NCT02485730). All participants provided written informed consent, as approved by the Ethics Committee. The study was performed in accordance with the principles of the Declaration of Helsinki.

### Biomarker Measurements and Classification

#### CSF and Plasma

Sample collection and processing were conducted according to standardized protocols, as previously detailed.^23^ CSF Aβ42/40 was measured with the exploratory NeuroToolKit, a panel of robust prototype immunoassays (Roche Diagnostics International Ltd., Rotkreuz, Switzerland), on a Cobas e601 module. CSF p-tau181 and t-tau (both corresponding to the mid-region domain of tau protein) were measured using the electrochemiluminescence Elecsys Phospho-Tau (181P) CSF and Elecsys Total-Tau CSF immunoassays, respectively, on a fully automated Cobas e601 module (Roche Diagnostics International Ltd.). CSF p-tau181/Aβ42 was measured using the Elecsys electrochemiluminescence immunoassay on a fully automated Cobas e601 analyzer (Roche Diagnostics International Ltd.). All CSF biomarker measurements were determined at the Clinical Neurochemistry Laboratory at the University of Gothenburg, Sweden. Blood samples were obtained on the same day as the LP. Eli Lilly and Company provided the measurements of the previously published in-house assay for plasma p-tau217 using the Meso Scale Discovery platform.^24^

#### PET

Participants underwent Aβ PET ([¹⁸F]flutemetamol) imaging following a cranial CT scan for attenuation correction, performed on a Biograph mCT scanner (Siemens Healthcare, Erlangen, Germany) at Hospital Clinic, Barcelona. PET data acquisition, 90 minutes after injection, lasted 20 minutes (4 frames of 5 minutes each). Images were reconstructed in 4 × 5-minute frames using the 3-dimensional Ordered Subset Expectation Maximization algorithm, incorporating both time-of-flight information and point spread function modeling. Aβ PET scans were quantified in Centiloid units using a validated in-house processing pipeline.^25^ Centiloid values were derived from the mean uptake in the standard Centiloid cortical target region,^26^ using the whole cerebellum as the reference region, based on previously established transformation parameters.

#### Biomarker Classification

According to previous work in the ALFA+ cohort, the AT(N) classification system was applied to define individual CSF biomarker profiles in accordance with the NIA-AA research framework for characterizing the AD neuropathologic processes.^1^ Fluid biomarker classification was determined using previously validated CSF cutoffs in the ALFA+ cohort: Aβ positivity (A+) was defined by an Aβ42/40 ratio <0.071, tau positivity (T+) by p-tau181 >24 pg/mL, and neurodegeneration positivity ([N]+) by t-tau >300 pg/mL.^23^

### Neuropsychological Assessment

#### MMSE

The Mini-Mental State Examination (MMSE) is a brief assessment of cognitive function. The Spanish version was used to evaluate temporal/spatial orientation, memory, attention, language, and constructive praxis.^27^

#### mPACC

The PACC is a validated measure of Aβ-related cognitive decline, pooling 4 z-transformed neuropsychological scores.^12^ In this study, we used the mPACC, which was developed for optimization and involves a semantic fluency measurement instead of the MMSE, enhancing sensitivity to early cognitive changes.^13^ The Free and Cued Selective Reminding Test, Spanish version, was used to assess verbal episodic memory with a controlled learning procedure, considering the total immediate recall (0–48).^28^ The Logical Memory subtest in the Wechsler Memory Scale-IV, Spanish version, was used to assess narrative episodic memory, considering the total delayed recall (0–50).^29^ The Coding subtest in the Wechsler Adult Intelligence Scale-IV, Spanish version, was used to assess processing speed and attention (0–135).^30^ The Semantic Fluency Test, Spanish version, was used to assess executive control through semantic verbal fluency (number of correct lexical items produced in 1 minute within the category “animals”).^31^

#### SCD-Q

The Subjective Cognitive Decline Questionnaire (SCD-Q) is a validated instrument designed to assess and quantify subjective cognitive decline (SCD). It includes an identical set of questions administered to both participants, “My-Cognition,” and their study partners, “Their-Cognition” (0–24 each version).^32^

### Statistical Analysis

The primary objective of the study was to identify longitudinal associations across the full sample, including Aβ-positive and Aβ-negative individuals. Sensitivity analyses evaluated these associations stratifying by Aβ status. A general description is provided further; full details are available in eMethods.

#### Definition of the Neuropsychological Outcome

The mPACC score was calculated using previously validated methods used for defining sensitive AD biomarker–based cognitive performance.^33,34^ In brief, the BL neuropsychological performance of the Aβ-negative group was used as reference, considering its mean and standard deviation to compute z-scores for each of the cognitive variables included in the mPACC. These AD biomarker–based cognitive z-scores were averaged to compute the mPACC. Consequently, mPACC scores, normalized to the BL measurements, reflected deviation from the robust control group defined by normal Aβ levels. The mPACC z-scores were computed at BL, follow-up (FU), and the corresponding longitudinal discrepancy between visits (FU-BL).

#### Definition of the Neuroimaging Outcome

Voxel intensities from Aβ PET images, referenced to the whole cerebellum and normalized to Montreal Neurological Institute space, were extracted for both BL and FU visits. To ensure robust longitudinal calculations, a mask including only voxels with nonzero values in both BL and FU scans was used. For each voxel in a participant's image, the longitudinal discrepancy was computed as the normalized percentage of change relative to the BL measurement. The resulting voxel-wise longitudinal discrepancy maps were smoothed using an 8-mm full-width at half-maximum Gaussian kernel.

#### Statistical Modeling

The longitudinal relationship of plasma and CSF biomarkers with cognitive function was evaluated using linear mixed-effects (LME) regression models (R software, version 4.2.1, RStudio, version 2022.07.1., lme4 package for R). The mPACC was treated as the outcome in independent LME models using fluid biomarkers (log10 transformed) as main predictors of interest. LME models considered 2 time points (BL and FU, for both outcomes and main predictors); used a random intercept for each participant; and were adjusted for BL age, sex, BL education, APOE ε4, and time interval between BL and FU visits (years), with time interactions included for all predictors. Standardized β coefficients with 95% CIs, *p* values, marginal and conditional *R*^2^ coefficients of determination (representing the variance explained by fixed effects as well as the combined fixed and random effects, respectively), and the Akaike Information Criterion were reported. In LME models, nominal *p* values <0.05 were considered statistically significant.

The relationship between PET imaging and cognitive function was evaluated using voxel-wise linear regression models (MATLAB software, version 2022b, Statistical Parametric Mapping [SPM], version 12). The Aβ PET longitudinal discrepancy was treated as the outcome in voxel-wise linear regression models, with the mPACC longitudinal discrepancy as the main predictor of interest, adjusted for BL age, sex, BL education, APOE-ε4, total intracranial volume, and time interval between BL and FU visits (years). As required by SPM, voxel-wise brain images were modeled as outcomes because of their 3-dimensional spatial complexity. Voxel-wise regression models aimed to assess associations rather than imply causality, under the premise that Aβ burden contributes to cognitive decline. Whole-brain voxel-wise PET imaging models used a gray matter mask excluding the cerebellum. In voxel-wise models, nominal *p* values <0.005, accounting for a cluster-extent correction >100 voxels, were considered statistically significant.

#### Sensitivity Analysis

The sensitivity analysis, stratified by Aβ-positive and Aβ-negative groups, evaluated the longitudinal association of plasma p-tau217, CSF p-tau181/Aβ42, and Aβ PET with mPACC scores, using the same models and equations previously described for the main analysis within each biomarker modality.

### Data Availability

The data that support the findings of this study are available from the corresponding authors, O.G.-R. or G.S.-B., on reasonable request.

## Results

### Characteristics of the Participants

The ALFA parent cohort comprised 2,743 CU middle-aged individuals, enriched for AD risk considering family history. Specifically, 47% had at least 1 parent diagnosed before age 75, and 86% had a parental history regardless of age at onset. The ALFA parent cohort had a high prevalence of APOE-ε4 carriers (35%). The nested ALFA+ cohort included a subset of 419 CU individuals, who were selected based on their AD risk profile, considering parental AD history and APOE-ε4.

In this study, 350 CU individuals from the ALFA+ cohort were included. This sample underwent comprehensive clinical and neuropsychological assessments (n_BL_ = 350; n_FU_ = 350), CSF sampling (n_BL_ = 350; n_FU_ = 264), blood sampling (n_BL_ = 349; n_FU_ = 338), and PET imaging (n_BL_ = 289; n_FU_ = 189). BL characteristics of the full sample and division by Aβ status are provided in Table 1. According to BL CSF biomarker levels, 228 individuals (65%) were classified as Aβ-negative and 122 (35%) were classified as Aβ-positive. In the full sample, the mean age was 61 years, 60% were female, the mean education was 14 years, and 55% were APOE-ε4 carriers. Significant BL differences were found between Aβ-positive and Aβ-negative groups: the Aβ-positive group was slightly older (*p* < 0.001), had a higher percentage of APOE-ε4 carriers (*p* < 0.001), and showed greater biomarker abnormalities, as measured with continuous plasma p-tau217 concentration (*p* < 0.001), CSF p-tau181/Aβ42 ratio (*p* < 0.001), and global Aβ PET load in Centiloid units (*p* < 0.001). No significant BL differences were found in sex, education, mPACC score, self-reported SCD score, study partner SCD score, and MMSE score.

**Table 1:** Baseline Characteristics of the Participants

	Full sample (n = 350)	Aβ-negative (n = 228)	Aβ-positive (n = 122)	*p* Value
Demographic				
Age, mean (SD)	60.93 (4.72)	60.28 (4.49)	62.13 (4.91)	<0.001
Sex (female), n (%)	211 (60.29)	138 (60.53)	73 (59.84)	0.991
Education, mean (SD)	13.53 (3.48)	13.64 (3.41)	13.30 (3.62)	0.383
Genetic				
APOE-ε4 (carriership), n (%)	191 (54.57)	98 (42.98)	93 (76.23)	<0.001
Biomarker				
Plasma p-tau217 (pg/mL), mean (SD)	0.153 (0.090)	0.136 (0.086)	0.185 (0.088)	<0.001
CSF p-tau181/Aβ42 (hybrid ratio), mean (SD)	0.014 (0.011)	0.010 (0.002)	0.023 (0.015)	<0.001
[^18^F]Flutemetamol PET (Centiloid units), mean (SD)	3.523 (17.249)	−4.125 (6.547)	17.403 (21.514)	<0.001
Neuropsychology				
PACC, mean (SD)	−0.01 (0.67)	0.00 (0.66)	−0.02 (0.70)	0.795
SCD-Q: participant, mean (SD)	4.11 (4.38)	3.93 (4.35)	4.44 (4.44)	0.308
SCD-Q: partner, mean (SD)	1.53 (2.47)	1.41 (2.45)	1.75 (2.50)	0.224
MMSE, mean (SD)	29.17 (0.95)	29.14 (0.91)	29.22 (1.02)	0.450

Abbreviations: Aβ = β-amyloid; ALFA+ = Alzheimer's and Families+; ANOVA = analysis of variance; BL = baseline; MMSE = Mini-Mental State Examination; mPACC = modified PACC; PACC = Preclinical Alzheimer's Cognitive Composite; p-tau = phosphorylated tau; SCD-Q = Subjective Cognitive Decline Questionnaire.

Results presented correspond to the mean and SD for continuous variables, as well as the number of observations and percentage (%) for categorical variables (reference level). The first column of participants corresponds to the full sample, and the second and third columns represent the division by Aβ status. Nominal *p* values from univariate ANOVAs (for continuous variables) and χ^2^ tests (for categorical variables) assessing BL characteristics between Aβ-negative and Aβ-positive groups were reported. mPACC was calculated as AD biomarker–based neuropsychological performance, using the BL Aβ-negative group as reference. The previously validated CSF biomarker cutoff used to define Aβ positivity in the ALFA+ cohort study was <0.071 for the Aβ42/40 ratio. The presence of subjective cognitive decline was quantified with the total score of the SCD-Q, using parallel questionnaire versions for the participant and the study partner.

### Longitudinal Association of Plasma p-Tau217 and CSF p-Tau181/Aβ42 With mPACC Score

**Figure 1:**
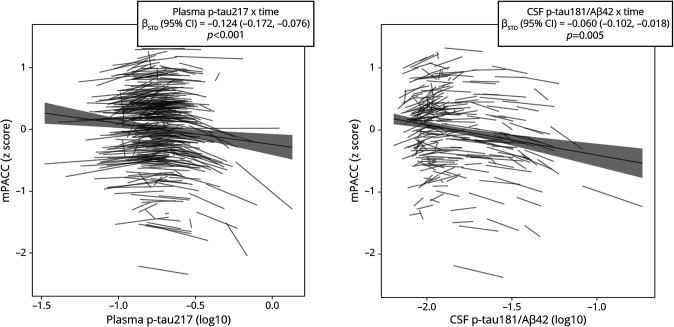
Longitudinal association of Plasma p-Tau217 and CSF p-Tau181/Aβ42 With mPACC Plots showing the predicted longitudinal relationship of plasma and CSF biomarkers with mPACC using LME models adjusted for age, sex, education, APOE-ε4, and longitudinal time interval, including time interactions with all predictors and a random intercept for each participant. The biomarker × time interactions predicting mPACC are shown on the top right side of each plot. Aβ = β-amyloid; LME = linear mixed-effect; mPACC = modified Preclinical Alzheimer's Cognitive Composite; p-tau = phosphorylated tau.

In the full sample, significant longitudinal associations with mPACC score were observed for plasma p-tau217 and CSF p-tau181/Aβ42. Results are presented in Table 2 and Figure 1; full details are given in eTable 1. In the model using plasma biomarker data, the time interaction indicated that longitudinal increments in the plasma p-tau 217 concentration were associated with longitudinal mPACC decline (β_STD_ = −0.124, 95% CI −0.172 to −0.076; *p* < 0.001). Similarly, in the model using CSF biomarker data, the time interaction indicated that longitudinal increments in the CSF p-tau181/Aβ42 ratio were associated with longitudinal mPACC decline (β_STD_ = −0.060, 95% CI −0.102 to −0.018; *p* = 0.005).

**Table 2:** Longitudinal Association of Plasma p-Tau217 and CSF p-Tau181/Aβ42 With mPACC

Outcome: predictor	n	Standardized β coefficient (95% CI)	*p* Value	*R* ^2^	AIC
mPACC: plasma p-tau217	349/338			0.274/0.825	1,069.507
Plasma p-tau217		−0.108 (−0.179 to −0.037)	0.003		
Time		0.064 (−0.017 to 0.144)	0.121		
Plasma p-tau217 × time interaction		−0.124 (−0.172 to −0.076)	<0.001		
mPACC: CSF p-tau181/Aβ42	350/264			0.274/0.823	989.733
CSF p-tau181/Aβ42		−0.081 (−0.169 to 0.007)	0.070		
Time		−0.011 (−0.086 to 0.063)	0.768		
CSF p-tau181/Aβ42 × time interaction		−0.060 (−0.102 to −0.018)	0.005		

Abbreviations: Aβ = β-amyloid; AIC = Akaike Information Criterion; BL = baseline; FU = follow-up; LME = linear mixed-effect; mPACC = modified Preclinical Alzheimer's Cognitive Composite; p-tau = phosphorylated tau

Results from LME models indicating number of observations (n) at BL/FU, standardized β coefficients, 95% CIs, nominal *p* values, the marginal and conditional *R*^2^, and AIC measurements. LME models were adjusted for age, sex, education, APOE-ε4, and time, including a time interaction for each predictor and a random intercept for each participant.

### Longitudinal Association Between Aβ PET and mPACC Score

**Figure 2:**
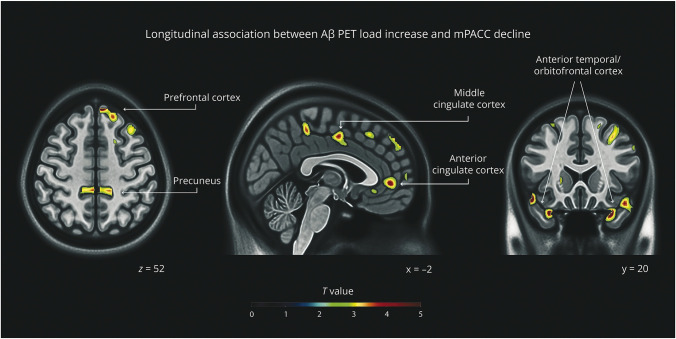
Longitudinal Association Between Aβ PET and mPACC Brain maps showing the longitudinal association between Aβ PET load increase and mPACC decline resulting from the voxel-wise linear regression model. The model was adjusted for age, sex, education, APOE-ε4, total intracranial volume, and longitudinal time interval. Results shown at nominal *p* values <0.005 and a cluster-size threshold correction >100 voxels. Aβ = β-amyloid; mPACC = modified Preclinical Alzheimer's Cognitive Composite.

In the full sample, significant longitudinal associations with mPACC score were observed for Aβ PET. Results are presented in Table 3 and Figure 2; full details are shown in eFigures 2–4. Longitudinal increases in the Aβ PET load in the prefrontal cortex, cingulate cortex, precuneus, and anterior temporal regions, extending slightly into the orbitofrontal cortex, were associated with longitudinal mPACC decline. The opposite contrast did not yield significant results.

**Table 3:** Longitudinal Association Between Aβ PET and mPACC

Outcome: predictor	n	Anatomical locations	Cluster-level		Peak-level		Peak MNI coordinates		
			k	*p* Value	T	*p* Value	x	y	z
Aβ PET: mPACC	189								
		Superior frontal cortex right	1,376	<0.001	4.600	<0.001	30	62	20
		Superior temporal pole right	362	0.010	4.410	<0.001	30	20	−30
		Superior temporal pole left	411	0.006	4.176	<0.001	−50	20	−16
		Precuneus right	319	0.014	3.897	<0.001	10	−40	56
		Anterior cingulate cortex left	347	0.011	3.792	<0.001	−2	46	0
		Superior frontal cortex left	149	0.077	3.691	<0.001	−26	54	28
		Middle cingulate cortex left	235	0.031	3.673	<0.001	−2	−6	46
		Posterior cingulate cortex right	143	0.082	3.216	0.001	8	−48	24
		Middle temporal pole left	136	0.089	3.189	0.001	−18	12	−42

Abbreviations: Aβ = β-amyloid; MNI = Montreal Neurological Institute; mPACC = modified Preclinical Alzheimer's Cognitive Composite.

Voxel-wise linear regression model results showing the longitudinal association between Aβ PET load increase and mPACC decline. The model was adjusted for age, sex, education, APOE-ε4, total intracranial volume, and longitudinal time interval. Results shown at nominal *p* values <0.005 and a cluster-size threshold correction >100 voxels.

### Stratification by Aβ Status

The sensitivity analyses stratified the sample by Aβ status, evaluating the longitudinal association of plasma p-tau217, CSF p-tau181/Aβ42, and Aβ PET with mPACC score. Results are presented in Tables 4 and 5; full details are given in eTables 2 and 3.

**Table 4:** Stratification by Aβ-Positive and Aβ-Negative Groups: Longitudinal Association of Plasma p-Tau217 and CSF p-Tau181/Aβ42 With mPACC

Group; outcome: predictor	n	Standardized β coefficient (95% CI)	*p* Value	*R* ^2^	AIC
Aβ-positive group					
mPACC: plasma p-tau217	122/118			0.418/0.821	434.674
Plasma p-tau217		−0.166 (−0.291 to −0.040)	0.010		
Time		−0.033 (−0.194 to 0.127)	0.682		
Plasma p-tau217 × time interaction		−0.121 (−0.194 to −0.049)	0.001		
mPACC: CSF p-tau181/Aβ42	122/92			0.411/0.802	401.773
CSF p-tau181/Aβ42		−0.153 (−0.294 to −0.013)	0.033		
Time		−0.133 (−0.315 to 0.050)	0.153		
CSF p-tau181/Aβ42 × time interaction		−0.044 (−0.124 to 0.037)	0.287		
Aβ-negative group					
mPACC: plasma p-tau217	227/220			0.205/0.850	671.084
Plasma p-tau217		−0.054 (−0.137 to 0.028)	0.197		
Time		0.110 (0.011 to 0.208)	0.030		
Plasma p-tau217 × time interaction		−0.084 (−0.157 to −0.011)	0.024		
mPACC: CSF p-tau181/Aβ42	228/172			0.211/0.852	633.327
CSF p-tau181/Aβ42		−0.004 (−0.083 to 0.076)	0.926		
Time		0.053 (−0.028 to 0.133)	0.201		
CSF p-tau181/Aβ42 × time interaction		0.003 (−0.050 to 0.057)	0.904		

Abbreviations: Aβ = β-amyloid; AIC = Akaike Information Criterion; BL = baseline; FU = follow-up; LME = linear mixed-effect; mPACC = modified Preclinical Alzheimer's Cognitive Composite; p-tau = phosphorylated tau

Results from LME models indicating the number of observations (n) at BL/FU, standardized β coefficients, 95% CIs, nominal *p* values, the marginal and conditional *R*^2^, and AIC measurements. LME models were adjusted for age, sex, education, APOE-ε4, and time, including a time interaction for each predictor and a random intercept for each participant.

**Table 5:** Stratification by Aβ-Positive and Aβ-Negative Groups: Longitudinal Association Between Aβ PET and mPACC

Group; outcome: predictor	n	Anatomical locations	Cluster-level		Peak-level		Peak MNI coordinates		
			k	*p* Value	T	*p* Value	x	y	z
Aβ-positive group									
Aβ PET: mPACC	78								
		Superior temporal pole left	140	0.057	4.24	<0.001	−40	10	−22
Aβ-negative group									
Aβ PET: mPACC	111								
		Superior frontal cortex right	442	0.001	4.291	<0.001	16	38	56
		Superior frontal cortex right	154	0.038	4.041	<0.001	24	−10	74
		Middle frontal cortex right	175	0.028	3.898	<0.001	32	46	38
		Middle frontal cortex left	191	0.023	3.798	<0.001	−30	22	56
		Middle frontal cortex right	205	0.019	3.782	<0.001	32	22	46
		Precuneus left	126	0.057	3.701	<0.001	−4	−40	76
		Postcentral left	138	0.047	3.694	<0.001	−62	−16	36
		Superior parietal cortex right	128	0.055	3.576	<0.001	44	−46	60
		Precuneus right	312	0.005	3.491	<0.001	6	−68	46
		Superior frontal cortex left	102	0.083	3.487	<0.001	−20	−2	68
		Inferior parietal cortex left	181	0.026	3.160	0.001	−38	−44	50

Abbreviations: Aβ = β-amyloid; MNI = Montreal Neurological Institute; mPACC = modified Preclinical Alzheimer's Cognitive Composite.

Voxel-wise linear regression model results showing the longitudinal association between Aβ PET load increase and mPACC decline. The model was adjusted for age, sex, education, APOE-ε4, total intracranial volume, and longitudinal time interval. Results shown at nominal *p* values <0.005 and a cluster-size threshold correction >100 voxels.

Among Aβ-positive individuals, significant longitudinal associations with mPACC score were observed for plasma p-tau217 and Aβ PET, but not for CSF p-tau181/Aβ42. In the model using plasma biomarker data, the time interaction indicated that longitudinal increments in the plasma p-tau217 concentration were associated with longitudinal mPACC decline (β_STD_ = −0.121, 95% CI −0.194 to −0.049; *p* = 0.001). In the model using CSF biomarker data, the time interaction indicated that longitudinal increments in the CSF p-tau181/Aβ42 ratio were not associated with longitudinal changes in mPACC score. In the model using voxel-wise PET imaging, longitudinal increase in the Aβ PET load in anterior temporal regions was associated with longitudinal mPACC decline. The opposite contrast did not yield significant results.

Among Aβ-negative individuals, significant longitudinal associations with mPACC score were observed for plasma p-tau217 and Aβ PET, but not CSF p-tau181/Aβ42. In the model using plasma biomarker data, the time interaction indicated that longitudinal increments in the plasma p-tau217 concentration were associated with longitudinal mPACC decline (β_STD_ = −0.084, 95% CI −0.157 to −0.011; *p* = 0.024). In the model using CSF biomarker data, the time interaction indicated that longitudinal increments in the CSF p-tau181/Aβ42 ratio were not associated with longitudinal changes in mPACC score. In the model using voxel-wise PET imaging, longitudinal increases in the Aβ PET load in the prefrontal cortex, precuneus, postcentral gyrus, and parietal cortex were associated with longitudinal mPACC decline. The opposite contrast did not yield significant results.

## Discussion

This prospective observational study evaluated longitudinal associations between multimodal early-changing core 1 AD biomarkers—plasma p-tau217, CSF p-tau181/Aβ42, and Aβ PET—and cognition, measured with the mPACC, over a 3-year period in CU individuals at increased risk of AD dementia. Main results revealed consistent longitudinal associations between multimodal core 1 AD biomarkers and cognition, highlighting the effect of AD pathologic burden accumulation on subtle cognitive decline. Plasma p-tau217 was a particularly sensitive marker of cognitive decline, reinforcing the strong potential of blood-based biomarkers for screening and monitoring in CU population. Voxel-wise Aβ PET imaging revealed region-specific associations linking early Aβ accumulation in key AD-related vulnerable regions to cognitive decline. Sensitivity analyses revealed that these associations already emerge during the Aβ-negative stage, further supporting the conceptualization of AD as a *continuum*. These findings align with previous studies linking AD biomarkers to cognitive performance in CU individuals,^3-5^ underscoring the importance of longitudinal monitoring using distinct biomarker modalities to delineate clinical-biological dynamics in the preclinical stage of AD.^19-21^

Fluid biomarkers analysis demonstrated significant longitudinal associations of plasma p-tau217 and CSF p-tau181/Aβ42 with cognition, highlighting the potential of minimally invasive blood-based biomarkers for tracking cognitive decline in the CU population. Findings showed that greater longitudinal increases in the plasma p-tau217 concentration and CSF p-tau181/Aβ42 ratio were associated with steeper longitudinal mPACC decline, as observed in independent models. These early-changing core 1 AD biomarkers demonstrated comparable predictive capacity in modeling subtle cognitive decline. These results align with previous research showing that plasma p-tau217 demonstrated strong prognostic associations with cognitive decline and clinical progression in CU individuals.^35-37^ This reinforces the recognition of validated plasma biomarkers as sensitive, accessible tools for tracking AD-related subtle cognitive decline before clinical onset. More broadly, the strong relationship between fluid biomarker changes and cognitive decline provides relevant insights for guiding preventive intervention strategies, supporting the use of these biomarkers for early monitoring of AD clinical-biological progression. Particularly, these findings emphasize the significance of assessing longitudinal dynamics beyond cross-sectional measures, with plasma p-tau217 emerging as a promising biomarker for subtle cognitive decline.

Neuroimaging analysis demonstrated significant longitudinal associations between Aβ PET and cognition, providing relevant insights into the brain regions most vulnerable to core 1 AD pathology in relation to subtle cognitive decline in the CU population. Findings showed that greater longitudinal increases in Aβ PET load in the prefrontal cortex, cingulate cortex, precuneus, and anterior temporal regions extending slightly into the orbitofrontal cortex were associated with longitudinal mPACC decline. These results aligned with previous research showing that Aβ PET burden was associated with longitudinal cognitive and functional decline across the AD *continuum*.^38^ This regional pattern of Aβ accumulation is consistent with early progression of Aβ pathology in key AD-related brain regions before clinical onset.^39^ These findings underscore the utility of voxel-wise PET imaging analysis in capturing longitudinal Aβ deposits with advanced spatial resolution, providing crucial complementary information to the analysis of soluble forms measured by fluid biomarkers, which change earlier in the AD *continuum* but lack information about the spatial localization of Aβ deposits. Longitudinal Aβ PET changes suggested critical brain regions for monitoring CU individuals at high risk of clinical impairment. The regional pattern of Aβ PET load accumulation observed also suggested a broader influence on brain structure and function, contributing to AD progression. Previous studies showed that Aβ pathology, measured in both CSF and PET, was associated with medial temporal lobe atrophy in CU individuals,^40,41^ a key predictor of clinical progression across the AD *continuum* (from CU to MCI and MCI to dementia).^42,43^ The spatial resolution of PET imaging is crucial for staging Aβ accumulation across brain regions at the intraindividual level and for distinguishing spatiotemporal patterns of Aβ accumulation linked to distinct clinical manifestations at the interindividual level, with potential relevance for personalized medicine approaches.^44^

Sensitivity analysis, stratifying the sample by Aβ status, provided additional insights into the longitudinal relationship between multimodal core 1 AD biomarkers and cognitive function in the CU population. Regarding fluid biomarkers, greater longitudinal increase in plasma p-tau217 concentration was associated with steeper longitudinal mPACC decline in both Aβ-positive and Aβ-negative groups. By contrast, no significant association was found between longitudinal increase in the CSF p-tau181/Aβ42 ratio and mPACC decline, stratified by Aβ status, underscoring the sensitivity of plasma p-tau217 to track very early subtle cognitive decline in aging and preclinical AD.^16,17^ Regarding neuroimaging, in the Aβ-positive group, greater longitudinal increase in the Aβ PET load in anterior temporal regions was associated with mPACC decline, whereas in the Aβ-negative group, greater longitudinal increases in frontoparietal regions were associated with mPACC decline, reflecting a pattern of Aβ PET accumulation similar to the one observed in the full sample, but more localized to early-accumulating areas. These regional patterns observed when stratifying by Aβ status provided further consistent detail into the progression of Aβ pathology in AD, in which frontoparietal brain regions begin to accumulate Aβ before temporal regions, moving from early to intermediate stages, respectively.^39^ Furthermore, these findings aligned with previous studies showing that Aβ subthreshold levels were associated with cognitive performance.^45,46^ Our study supports that subtle cognitive decline can be identified in relation to AD pathologic progression, as measured by core 1 AD biomarkers, even in the absence of Aβ positivity. The associations observed in the Aβ-negative range reinforce the conceptualization of AD as a clinical-biological *continuum*, rather than a discrete entity, as an essential perspective for advancing early detection and developing diagnostic tools grounded in longitudinal, integrative clinical-biological frameworks.^47^

Improving the clinical-biological characterization of preclinical AD is essential for enhancing early detection and prevention efforts because an estimated 45% of dementia cases may be preventable by addressing modifiable risk factors.^48^ Effective treatments, such as anti-Aβ therapies, are already available, with promising treatments targeting early disease stages expected in the coming years.^7^ In this context, our study suggests some implications for clinical trial designs considering selection criteria in anti-Aβ trials. Restricting enrollment to only Aβ-positive individuals may exclude a vulnerable population with low resilience to early AD pathology, particularly those who are Aβ-negative but already experiencing subtle cognitive decline linked to subthreshold Aβ accumulation. Previous studies supported that integrating biological and clinical data may improve diagnostic criteria, risk stratification, and preventive strategies for AD.^49^ Nonetheless, significant challenges remain in the field. Methodological differences, such as the variety of neuropsychological assessments and biomarker measurements, may contribute to inconsistencies across studies and limit the generalizability of findings.^21^

This study has both strengths and limitations. Among its strengths, while most previous investigations relied on single-modality, cross-sectional biomarker data, this study examined longitudinal associations between multimodal biomarkers and cognition. We conducted a longitudinal analysis using core 1 AD biomarkers, including distinct fluid and neuroimaging modalities to provide a comprehensive perspective on how distinct early biomarker changes are associated with subtle cognitive decline in the CU population, distinguishing our work from previous studies. In addition, we used the mPACC, encompassing multiple standardized neuropsychological tests, leveraging a validated, Aβ-sensitive measure of AD-related subtle cognitive decline, aiming to enhance generalizability of results. Defining the mPACC as a biomarker-based neuropsychological outcome, using the Aβ-negative group as reference, further strengthened the sensitivity of our analyses, as robust neuropsychological methods are critical to identify subtle cognitive decline.^33,34^ Finally, because Aβ changes earlier in CSF than in PET, our study using CSF to define Aβ status was well suited for identifying the earliest changes in aging and preclinical AD.

Regarding study limitations, we acknowledge the exclusive focus on core 1 AD biomarkers without including core 2 AD biomarkers (particularly tau PET imaging). At this early stage, with most of our sample being CSF Aβ-negative, it is challenging to determine the extent to which plasma p-tau217, an early-changing diagnostic core 1 AD biomarker, specifically reflects tau-related changes detectable through PET imaging, a late-changing prognostic core 2 AD biomarker.^2^ Although plasma p-tau217 is more strongly associated with Aβ pathology in early disease stages, we acknowledge that it may have captured emerging tau accumulation detectable through PET imaging, particularly in Aβ-positive individuals.^50^ Therefore, the associations characterized in this study may reflect not just Aβ-related processes but also downstream mechanisms, including emerging tau pathology and nonspecific processes involved in AD pathophysiology, such as neurodegeneration and inflammation. In addition, the associations characterized may also reflect overlapping processes, including copathologies such as vascular brain injury or alpha-synucleinopathy, warranting further investigation in relation to subtle cognitive decline.

In conclusion, this study highlights the significance of evaluating longitudinal multimodal core 1 AD biomarkers, including plasma p-tau217, CSF p-tau181/Aβ42, and Aβ PET, for tracking subtle cognitive decline in CU individuals. The findings underscore the complementary value of distinct biomarker modalities and reinforce the value of longitudinal clinical-biological characterization in aging and preclinical AD, crucial for early detection, monitoring, and intervention focused on the asymptomatic stage of the disease.

## Appendix Coinvestigators

**Table:**

Name	Location	Role	Contribution
Federica Anastasi	Barcelonaβeta Brain Research Center, Spain	ALFA investigator	Collection of data
Annabella Beteta	Barcelonaβeta Brain Research Center, Spain	ALFA data acquisition	Collection of data
Raffaele Cacciaglia	Barcelonaβeta Brain Research Center, Spain	ALFA investigator	Collection of data
Irene Cumplido Mayoral	Barcelonaβeta Brain Research Center, Spain	ALFA investigator	Data analysis
Alba Cañas	Barcelonaβeta Brain Research Center, Spain	ALFA data acquisition	Collection of data
Marta del Campo	Barcelonaβeta Brain Research Center, Spain	ALFA data acquisition	Collection of data
Carme Deulofeu	Barcelonaβeta Brain Research Center, Spain	ALFA data management	Database construction
Ruth Dominguez	Barcelonaβeta Brain Research Center, Spain	ALFA data acquisition	Study design
Maria Emilio	Barcelonaβeta Brain Research Center, Spain	ALFA data acquisition	Collection of data
Sherezade Fuentes	Barcelonaβeta Brain Research Center, Spain	ALFA data management	Database construction
Patricia Genius	Barcelonaβeta Brain Research Center, Spain	ALFA investigator	Data analysis
Armand González Escalante	Barcelonaβeta Brain Research Center, Spain	ALFA investigator	Collection of data
Laura Hernández	Barcelonaβeta Brain Research Center, Spain	ALFA data acquisition	Collection of data
Jordi Huguet	Barcelonaβeta Brain Research Center, Spain	ALFA data management	Database construction
Paula Marne	Barcelonaβeta Brain Research Center, Spain	ALFA data acquisition	Collection of data
Tania Menchón	Barcelonaβeta Brain Research Center, Spain	ALFA data acquisition	Collection of data
Paula Ortiz	Barcelonaβeta Brain Research Center, Spain	ALFA data acquisition	Study design
Wiesje Pelkmans	Barcelonaβeta Brain Research Center, Spain	ALFA investigator	Data analysis
Albina Polo	Barcelonaβeta Brain Research Center, Spain	ALFA data acquisition	Collection of data
Sandra Pradas	Barcelonaβeta Brain Research Center, Spain	ALFA data acquisition	Collection of data
Blanca Rodríguez Fernández	Barcelonaβeta Brain Research Center, Spain	ALFA investigator	Data analysis
Núria Tort-Colet	Barcelonaβeta Brain Research Center, Spain	ALFA investigator	Data analysis
Iman Sadeghi	Barcelonaβeta Brain Research Center, Spain	ALFA investigator	Data analysis
Anna Soteras	Barcelonaβeta Brain Research Center, Spain	ALFA study coordination	Collection of data
Marc Vilanova	Barcelonaβeta Brain Research Center, Spain	ALFA data acquisition	Collection of data
Natalia Vilor Tejedor	Barcelonaβeta Brain Research Center, Spain	ALFA investigator	Data analysis

## Glossary

Aβ: β-amyloid
AD: Alzheimer disease
ALFA: Alzheimer's and Families
ALFA+: Alzheimer's and Families+
BL: baseline
CU: cognitively unimpaired
FU: follow-up
LME: linear mixed-effect
MMSE: Mini-Mental State Examination
mPACC: modified PACC
NIA: National Institute on Aging
PACC: Preclinical Alzheimer's Cognitive Composite
p-tau: phosphorylated tau
SCD: subjective cognitive decline
SCD-Q: Subjective Cognitive Decline Questionnaire
SPM: Statistical Parametric Mapping
